# Associations between health literacy, cognitive function and general literacy in people with schizophrenia attending community mental health clinics in Australia

**DOI:** 10.1186/s12888-022-03901-7

**Published:** 2022-04-07

**Authors:** Sumana Thomson, Cherrie Galletly, Christopher Prener, Suzanne Garverich, Dennis Liu, Alisa Lincoln

**Affiliations:** 1 Northern Adelaide Local Health Network, Adelaide, South Australia; 2 Adelaide Medical School, The University of Adelaide, Adelaide, South Australia; 3 Ramsay Health Care (SA) Mental Health Services, Adelaide, South Australia; 4 Department of Sociology and Anthropology, Saint Louis University, St. Louis, MO USA; 5 Institute for Health Equity and Social Justice Research, Northeastern University, Boston, MA USA

**Keywords:** Social determinants, Aural literacy, Verbal literacy, Health education, Psychosis

## Abstract

**Background:**

Health literacy (HL) has been defined as the ability of individuals to access, understand, and utilise basic health information. HL is crucial to patient engagement in treatment through supporting patient autonomy, informed consent and collaborative care. In people with physical disorders, poor HL is associated with poor health outcomes, but less is known about HL in people with severe mental illness. This study aimed to assess HL and investigate the associations between education, cognitive function, general literacy, and HL in participants with schizophrenia attending community mental health clinics.

**Method:**

Fifty-two outpatients with schizophrenia attending a public community mental health clinic in Adelaide, Australia completed the Test of Functional Health Literacy in Adults—Short Form (S-TOFHLA) along with tests of cognition, aural and reading literacy and numeracy including Digit Symbol Coding (DSC), verbal fluency, the Wechsler Adult Intelligence Scale (WAIS-IV), Woodcock-Johnson III (Part 4 and 9) and the Lipkus numeracy scale. Sixty-one percent of participants were male. Participants had a mean age of 41.2 (SD 9.9) years and a mean of 11.02 (SD 1.5) years of education.

**Results:**

The majority of participants had very poor aural and verbal literacy and poorer literacy correlated with fewer years of education. On the S-TOFHLA, 81% of participants had adequate HL; 6% were marginal and 13% were inadequate. There was a positive correlation between education and HL, with those with more years of education scoring higher for HL. There was also a significant association between better HL and better working memory and attention.

**Conclusions:**

Consistent with previous research in schizophrenia, our participants had reduced educational attainment, aural and reading literacy and cognitive function compared to population norms. However, HL was better than expected given that previous research has found that people with psychiatric disorders tend to have lower HL, compared to the general population. This may reflect effective case management of our participants whilst attending the community clinics and supports ongoing research and intervention regarding HL in people living with mental illness.

## Introduction

In the past decade, increasing emphasis has been placed on patient engagement as a key component of high-quality, recovery-based mental health care [1]. While there are varying definitions, the core components of patient engagement can be considered to be self-efficacy, autonomy and empowerment [2]. This ability to engage in making and implementing decisions about health care is vital to informed consent, shared consent and personal recovery.

However, to achieve collaborative engagement, there are several key capabilities which can often be overlooked or assumed, including the ability to read (literacy), absorb oral information (aural literacy), and to have a basic understanding of functional health tasks such as reading and comprehending instructions about medication (health literacy) [3, 4]. For patients who have a limited understanding of basic health-related concepts, such as information about their diagnosis and treatment options, successfully navigating clinical encounters may be fraught with challenges. This vital gap may place patients at risk of errors in understanding information and instructions about their care, including treatment plans and medication. Poor HL can impede patient’s ability to receive and comprehend health information and fully participate in their care. This can result in an increased risk of poor health outcomes including medication errors, adverse events, non-concordance in therapy and disengagement from treatment.

### Health literacy

Health literacy (HL) has been defined as the degree to which individuals have the capacity to obtain, process, and understand basic health information [5]. This includes the capacity to understand information about their illness and to make decisions regarding medication and other treatment options. In recent years, HL has increasingly been recognized as a significant health concern and priority, with national and international campaigns created to identify and address this gap. HL is recognized as a core determinant of health, rooted in social inequity and required for empowered, active participation in health care [6, 7].

The prevalence of low health literacy in Australia has been found to be significant. In the Australian Bureau of Statistics 2006 Adult Literacy and Life Skills Survey, 60% of Australians were found to have low HL, with 59% of 17 to 74 years old participants described as possessing inadequate HL to effectively understand and apply health-related information in their lives [8]. Little national data on this scale has been obtained since, with only one recent Australian survey using Health Literacy Questionnaire (HLQ) finding 9% of participants disagreed or strongly disagreed that they could manage their health, with those with poorer reported health describing poorer HL [9]. A recent scoping review further emphasized the impact of limited HL, well-established to contribute to poorer health outcomes, on health service utilization, [10]. Deficits in HL have also been identified in Indigenous, culturally-diverse and remote communities with more studies needed to understand the nature and causes of HL and the relationships between psychosocial disadvantage and HL in these marginalized populations [11].

Inadequate HL has also been found internationally. In the United States, HL has been established to be an area of public health concern, with the 2003 National Assessment of Adult Literacy (NAAL) study finding approximately 36% of Americans surveyed to have basic or below basic HL [12]. A systematic review of 85 health literacy studies in the US found that over one quarter of subjects (26%) had low health literacy and one fifth had marginal health literacy, with lower HL being associated with being African American, poorer education and older age [13]. While this is consistent with more recent studies indicating low HL in areas of disadvantage, there have been few recent reviews in the prevalence of limited HL in the United States, highlighting the need for further study [14].

Similarly, in Europe, a recent large (*n* = 8000) survey of the EU found almost half (47%) had inadequate (insufficient or problematic) HL [15]. As with studies of the United States, the United Kingdom, Albania and Australia, inadequate HL was found to be concentrated in populations of socioeconomic disadvantage [15–17]. The social determinants of health such as socioeconomic status, education, race and social supports continue to play a powerful role in health outcomes. Socio-economically patterned disease cascades remain entrenched with the effects of transgenerational trauma and disadvantage in society, highlighting the need for the ‘social gradient’ for health to be considered in public health policy [15, 18]. There is evidence lower HL can play a mediating role in racial disparities and poor health outcomes, emphasising the need for an increased understanding of the interaction between race, social inequity and health systems in this vulnerable population [19].

### Health literacy and physical health

There is a considerable body of literature examining the relationship between health literacy and physical health. Studies of health literacy in people with physical disorders such as cardiovascular and metabolic disorders have demonstrated that individuals with poor health literacy have significantly worse health outcomes, including poorer engagement with preventative health measures, and greater morbidity and mortality [20, 21].

Marginal or inadequate HL has been found to be an independent predictor of all-cause mortality and cardiovascular death [22]. Incorrect use of medications due to poor health literacy places individuals at risk of serious medication errors [22]. Health literacy has also been found to be associated with differential use of health care services. Patients with low HL are more likely to visit emergency departments, have more admissions, and are less likely to follow treatment plans [23–26].

### Health literacy and schizophrenia

The risks for poor participation in health care and poorer health outcomes related to low HL are particularly concerning for people with serious mental illness (SMI). In addition to psychiatric symptoms and cognitive impairment, schizophrenia is associated with significant metabolic comorbidity with an increased risk of diabetes, obesity and cardiovascular disease, which are known to cause premature morbidity and mortality [27]. The second Australian survey of psychosis, a study of 1825 participants, found over half of participants met the criteria for metabolic syndrome [28]. The same study found that cardiometabolic comorbidity in people living with psychosis was under-investigated and under-treated despite relatively high rates of attendance at primary care physicians [28, 29]. People with SMI are also likely to be living in disadvantaged circumstances [30].

### Current relevance of health literacy

The relevance of health literacy as a vital capability has never been more evident. The COVID-19 pandemic has cast a spotlight on the need for effective public health communication, screening and vaccination. Recent literature has highlighted the importance of health literacy to enable individuals and communities to be able to engage with vital health messaging, including lockdown restrictions, social distancing and vaccinations [31, 32]. Further, those with schizophrenia are left highly vulnerable due to poor mental health, cognitive impairment, social exclusion, limited internet skills and physical morbidity [33]. Recent evidence has also emerged of significantly higher rates of mortality from COVID-19 in those with schizophrenia, highlighting the vital need for focus on HL in this group [34]. The City of Playford in Adelaide, the demographic of this study, is considered one of the most disadvantaged urban local governments areas in Australia, with a ten-year life expectancy gap compared to affluent regions [35]. The cumulative, multi-dimensional vulnerability to COVID-19 morbidity of populations with high levels of transgenerational trauma, social disadvantage and marginalisation with the associated elevated rates of physical and psychiatric comorbidity, have been described, highlighting the relevance of effective and accessible health messaging [36].

However, although it may be expected that people with poor health literacy might have more difficulty managing their illness and interacting with clinical services, there has been relatively little research into health literacy in those with SMI. Most research that has been undertaken has focused on mental health literacy (understanding relating to mental health concepts and diagnoses) rather than overall health literacy [37]. A recent systematic review of health literacy in people living with mental illness, identified a paucity of studies. This is complicated in interpretation by small studies and further limited by the use of a broad variety of measures which cannot be compared, ranging from functional HL assessments, such as the S-TOFHLA and The Rapid Estimate of Adult Literacy in Medicine (REALM), to multidimensional surveys [38].

Previous studies in America and China have explored health literacy in people with depression, finding that better HL is associated with fewer depressive symptoms, but HL in those with psychotic disorders such as schizophrenia has rarely been investigated [39–42]. In a cross-sectional prevalence study of 40 clozapine patients, marginal or low HL was found in more than a quarter of the population using The Rapid Estimate of Adult Literacy in Medicine (REALM) [43]. This was consistent with two other studies of people with diverse psychiatric diagnoses on the REALM [44, 45]. By contrast, in Australia, a study of 30 participants with Major Depression and 30 with schizophrenia found health literacy in the adequate range for 93% of the Major Depression group and 97% of the schizophrenia group. Health literacy was positively correlated with education but not with medication adherence [46]. The authors commented that they found better levels of health literacy than expected compared to national rates of HL. Finally, it has been noted that there is a dearth of studies examining outcomes of HL in SMI such as the impact of HL on health service utilisation or civic engagement in the studied populations. This would benefit from exploration as the literature on HL and SMI grows.

Given the paucity of studies in HL in SMI, particularly schizophrenia, and the significant level of physical morbidity and heightened risk of mortality during the COVID-19 pandemic, the need for research in this group has never been more critical. In this study we examined HL in people living with schizophrenia to add to this limited literature base and highlight the importance of HL in those living with SMI. In this study, we evaluated functional health literacy including prose comprehension and numeracy in patients with schizophrenia to examine the extent of and factors associated with poor HL in schizophrenia, with the aim for this information to be of benefit in providing effective clinical care and public health policy in this vulnerable population.

## Methods

Community mental health teams in Adelaide, Australia, provide multi-disciplinary assessment and follow up to people with a range of mental health conditions, including mood disorders, severe personality disorders and psychotic disorders, predominantly schizophrenia and schizoaffective disorder. Those attending community clinics receive support through a combination of psychiatric outpatient clinics with psychology, occupational therapy, social work and care coordination where relevant, as well as attending specific nurse-led clinics for depot antipsychotic medication and clozapine monitoring. All services are delivered at no cost to the those attending.

The participants in our study were recruited from people with mental illness who made clinical contact with the Playford Community Mental Health Team during the period of 16/04/2014 to 14/05/2014, as a part of a larger international study of heath literacy and mental health stigma, levels of shame and discrimination, health service utilisation and civic engagement funded by the National Institute for Mental Health (NIMH). Patients were offered study information and invited to participate. Inclusion criteria included both voluntary patients and those on community mental health treatment orders and included any form of contact (crisis calls, regular or acute outpatient psychiatric reviews or multidisciplinary contact).

A total of 199 people contacted the team during the recruitment period. Research staff discussed the study with them, and potential participants who were not fit to give informed consent, had severe thought disorder, or did not have sufficient competence in English to complete the study instruments, were not included Their treating clinicians were informed that this discussion had taken place. There were 101 people enrolled. The study was approved by the Queen Elizabeth Hospital, Adelaide, SA Human Ethics Committee and all participants provided written informed consent. Of the sample, 52 participants had a clinical diagnosis of schizophrenia and were therefore included in this study.

Their mean age was 41.2 (SD 9.9) years and 61% were male. More than half (53.8%) lived alone, 30.7% with family or spouse and 13.4% in a group setting or supported accommodation. The majority (96%) received welfare benefits and 75% were unemployed (Table 1).

**Table 1:** Participant characteristics

Variable	Schizophrenia: *n* = 52: n(%) or mean (SD)
**Age (y), mean (SD)**	41.2 (9.9)
**Gender, n (%)**	
Male	61
Female	39
**Employment status, n (%)**	
Employed	25
Unemployed	75
**Accommodation, n (%)**	
Lived alone	53.8
Lived with family	30.7
Lived in group setting or supported	13.4
**Welfare benefits, n (%)**	96
**Education (y), mean (SD)**	11.02 (1.5)

The study was administered by trained research staff with an iPad-based questionnaire that covered a range of topics. This assessment took place separately from their appointments with clinicians. Initial sections of the interview included demographic questions and the Test of Functional Health Literacy in Adults – Short Form (S-TOFHLA), a well-validated tool for assessing health literacy [47]. Scores in the S-TOFHLA range from 0 to 36, with participants assessed as having adequate HL (> 23), marginal (17–22) or inadequate HL (0–16). Two parts of the Woodcock-Johnson III measuring aural literacy (Part 4 or WJ4) and reading literacy (Part 9 or WJ9) were administered [48, 49]. WJ4 evaluated aural literacy by having respondents follow pre-recorded instructions to point to different items in a given set of pictures. WJ9, which assessed reading literacy, asked respondents to identify the correct words to complete phrases. The WJ4 and WJ9 are expressed as raw age-adjusted, grade-equivalent standardised scores. The Lipkus numeracy test, expressed as an un-normed score, was used to measure numeracy by assessing how well participants could evaluate percentages and proportions [50].

Further neurocognitive data were collected using Wechsler Adult Intelligence Scale (WAIS-IV) Digit Symbol Coding (DSC) test, a validated measure, expressed as a normed score, assessing executive functioning and processing speed, and Verbal Fluency (VF; animal naming) which additionally assesses verbal ability and is expressed as a z-score, normative, stratified by age and education [51, 52]. The consent discussion generally took about 10 min and the study interviews generally took about 2 h (with breaks). The diagnosis made by the treating psychiatrist was obtained from the case notes. Data analysis was performed with Stata v14 (Stata Corp 2015) and R (R Core Team 2019). Correlations between years of education and results on S-TOFHLA and the cognitive tests were analyzed by Pearson correlations with a *p* value of < 0.05 taken to indicate significance. Participants were advised that once the study was completed, results would be made available to them on request.

## Results

Using the S-TOFHLA, 81% of all participants with schizophrenia met criteria for adequate health literacy; 6% were marginal and 13% were inadequate. Participants had a mean of 11.02 (SD 1.5) years of education. Ninety percent of the study population were at or below 8th grade (Year 8) level for aural literacy (WJ4), and 63% were at or below 8th grade (Year 8) for reading literacy (WJ9). Study participants had lower scores on the WJ9 than population norms, measuring reading literacy (mean 8.3, SD 4.5). These findings underscore a key divide in our data between adequate health literacy, which a majority of our respondents have, and limited reading and aural literacy. Participants also had limited numeracy, with 40% (SD 0.495) answering fewer than half of the Lipkus numeracy scale questions correctly. Beyond these findings, participants’ performance on measures of cognitive functioning, including the WAIS-IV DSC (mean 7.20; SD 2.06) and verbal fluency (z-score -0.46; SD 1.27) suggest that our participants have impaired performance across a range of cognitive abilities (Table 2).

**Table 2:** Health literacy (S-TOFHLA), aural literacy (WJ4), reading literacy (WJ9), cognitive functioning (WAIS-IV digit symbol coding and verbal fluency), and numeracy (Lipkus) results

Measure (Range)	Result (%) or mean (SD)
**S-TOFHLA (0-100**^a^**), n (%)**	
0–16 (inadequate)	13
17–22 (marginal)	6
23–36 (adequate)	81
**WJ4 (aural literacy; 0.01–10.7**^a^**)**	4.5 (0.252)
**WJ9 (reading; 1.6–19.0**^a^**)**	8.3 (4.5)
**WAIS IV-DSC (3.0–12.0**^a^**)**	7.20 (2.06)
**WAIS IV-VF (-3.0–3.4**^a^**)**	-0.46 (1.27)
**Lipkus (numeracy; 1-11**^a^**)**	0.391 (0.006)

^a^S-TOFHLA and Lipkus scores represent the total number of correct answers. WJ4 and WJ9 scores represent grade equivalent scores, with 1 corresponding to first-grade level. DSC and VF values represent normed scores. DSC scores are means based on age and VF scores are z-scores based on age and years of education

There was a moderate, positive relationship (0.359; *p* < 0.05) between years of education and the S-TOFHLA score, with those with more years of education scoring higher on HL. There was also a positive correlation between the S-TOFHLA score and the aural and reading literacy scores, and to a lesser extent, DSC scores (Table 3).

**Table 3:** Correlations between health literacy, years of education, aural and reading literacy, numeracy, and WAIS-IV digit symbol coding (means) and verbal fluency (z-score) in people with schizophrenia

		**Correlation with years education (*n* = 49)**		**Correlation with S-TOFHLA (HL) (*n* = 43)**	
Test	Means (SD)	Correlation coefficient	*P* value	Correlation coefficient	*P* value
WJ4 (aural literacy)	0.933 (0.252)	0.247	0.088	0.511	0.0005
WJ9 (reading)	0.650 (0.481)	0.180	0.215	0.508	0.0005
WAIS IV-DSC	7.200 (2.057)	-0.011	0.938	0.381	0.012
WAIS IV-VF	-0.460 (1.265)	0.049	0.737	0.240	0.122
Lipkus (numeracy)	0.404 (0.495)	0.391	0.006	0.528	0.0003

## Discussion

Several important conclusions emerged from this study. Firstly, the majority of participants with schizophrenia had very poor aural and reading literacy, scoring at or below eighth grade level despite a mean of eleven years of schooling. This is consistent with findings of deficits in education attainment in studies of prodromal psychosis and established schizophrenia, with consequent social and vocational disadvantage [53, 54].

Participants were predominantly single and unemployed. While this is consistent with national data gathered in a psychosis population [29], these rates are higher than the general population in this region. This is likely reflective of the level of cognitive, social and functional impairment associated with schizophrenia, compounding disadvantage in this vulnerable group.

There were strong associations between HL and years of education, aural and reading literacy, and numeracy. This was expected as those struggling with basic literacy would be anticipated to experience difficulties interpreting health information. There was a weaker association with DSC, which measures working memory and attention, less proximal to literacy skills and no association with verbal fluency, which requires executive function. These findings indicate HL is more strongly associated with education, literacy and numeracy than with more general cognitive domains.

Finally, despite poor aural and verbal literacy, health literacy was higher than anticipated, with only 13% found to have inadequate health literacy. These findings suggest that, despite low levels of literacy and cognitive impairment, people with schizophrenia attending community mental health clinics generally do have adequate HL. This study supports a smaller previous study in a similar population [46], and adds to the limited evidence base on HL and SMI.

One possible explanation for the encouraging findings of adequate HL is the level of support provided to people with schizophrenia attending community mental health clinics in the northern suburbs of Adelaide. Many patients with chronic schizophrenia in community clinics receive a care coordination service. This involves an ongoing connection with one or more staff members in a team. Psychoeducation is a core component of care. Regular outpatient psychiatric appointments, care coordination and psychosocial rehabilitation, often provided in partnership with non-government agencies, allow for frequent contact and opportunity for discussion of physical health as well as support attending and engaging with health appointments.

Psychoeducation, long-recognised as a cornerstone of good psychiatric care, has been found to increase engagement and medication concordance and reduce stigma and shame, with studies showing its efficacy in reducing risk of relapse and readmission and reducing length of stay for inpatients [55]. Given decreased occupational and social engagement, poor verbal and aural literacy, these positive findings of health literacy may indicate that the clinicians supporting the participants in this study have been effective in providing ongoing education in managing their mental and physical disorders.

These findings also raise questions about the relationship between health literacy and fundamental elements of literacy including the ability to read, understand oral instructions, and work with numbers as well as the need for further attention to the measurement of these concepts particularly among people with serious mental illness. This is an area where additional research disentangling health literacy from these other aspects of literacy would be of benefit.

There are limitations in this study, including the relatively small sample size, lack of matched controls, and potential selection bias, as those choosing to participate in this study may have better insight and engagement. However, the use of a broad sample based on clinical contact, regardless of legal orders, may assist this sample in being more clinically representative. There are wider challenges within the study of HL, with multiple measures and definitions leading to difficulties assessing and establishing prevalence of this pressing public concern other than its convincing international scope [10]. With so few studies examining HL in SMI, the limited literature appears conflicting [43], or exploring different facets of HL [56], leading to pitfalls in comparison. This emphasizes the need for further research to establish the true extent and impact of HL in psychiatric populations.

Qualitative studies could provide rich insight into the experiences of people with limited HL and what factors are experienced as beneficial in improving HL. Further larger scale quantitative studies could examine the association between HL and multiple clinical domains relevant to health care provision and policy. These could include examining the association between HL and the treatment provided, such as whether those regularly attending clozapine or depot antipsychotic clinics have better HL. More broadly, there may be an association between the level of service provided and frequency of contact.

Studies examining the associations between HL and symptom severity of illness, cognition and level of functioning would be valuable to understanding HL in this population. The association of HL and duration of untreated psychosis could also be a valuable area for further research, particularly in young people with early psychosis. This could be of benefit to investigate the effects of psychosis on education attainment, general literacy, alongside the consequences of transgenerational trauma, underemployment and social disadvantage to inform development of targeted psychiatric and social interventions in this group [57]. Finally, another area for focus given the current paucity of literature, as highlighted by the recent systematic review, is the impact of low HL in those living with SMI on health care utilisation and civic engagement [38].

Based on these findings and from future research, recommendations can be made regarding need for awareness for HL in those with SMI. One recommendation may be the implementation of routine assessment of HL in people attending mental health care settings, particularly those with significant social disadvantage [58]. Routine evaluation using the S-TOFHLA, a validated and time-efficient tool, can be considered and targeted support and interventions for those with low HL arranged, including referral to specialised support to improve general literacy and numeracy. Collaboration with and education of community mental health care coordinators and leaders regarding HL, as well as other psychosocial support agency stakeholders such as non-government community organisations could be of benefit to identify and support those with limited HL. Education and direct engagement with people attending psychiatric care settings for their care about HL and the support available for this may also help to mitigate the dual stigma of limited literacy and SMI, which has previously been described [59]. Finally, advocacy to highlight and address the complex and entrenched layers of social disadvantage which often underpin low HL should also be considered integral in developing policy to address HL at a systemic level and remains central to developing policy in this area [60].

A focus on vulnerable and marginalised groups remains vital in examining and addressing low HL given the increased rates of physical morbidity and as well as mortality in the COVID-19 pandemic in those with schizophrenia. The heightened vulnerability of the socioeconomically disadvantaged communities during the COVID-19 pandemic, such as the population included in this study, remains concerning and would benefit from ongoing advocacy and policy to address the social and systemic factors of disadvantage and inequity underpinning this increased mortality and morbidity as well as interventions to support ongoing access to health care and services.

## Conclusions

Health literacy is a vital component in achieving patient engagement and offering high-quality, recovery-based mental health care. Our findings suggest that, at least in some people with serious mental illness, health literacy is surprisingly good. It would be important to identify the reasons for this, and to ensure that the factors contributing to this outcome can be made available for all people with serious mental illness. This is especially vital given the high rates of physical ill health with those with psychiatric morbidity and socioeconomic disadvantage, who remain particularly vulnerable in the COVID-19 pandemic.

## Data Availability

The data that support the findings of this study was collected as part of a larger study. The data are available from the Institute for Health Equity and Social Justice Research, Northeastern University, but restrictions apply to the availability of these data, which were used under license for the current study, and so are not publicly available. Data however may be available from the authors upon reasonable request and with permission of Northeastern University.
